# A Retrospective Study of Sildenafil Administration in 55 Cats with Cardiopulmonary Disease (2009–2021)

**DOI:** 10.3390/ani14152218

**Published:** 2024-07-31

**Authors:** Jasmine Huynh, Samantha Dolan, Emily T. Karlin, Lisa M. Freeman, Elizabeth A. Rozanski, John E. Rush

**Affiliations:** Department of Clinical Sciences, Cummings School of Veterinary Medicine, Tufts University, North Grafton, MA 01536, USA

**Keywords:** sildenafil, pulmonary hypertension, cardiomyopathy, congenital heart disease, cardiopulmonary disease, congestive heart failure

## Abstract

**Simple Summary:**

While sildenafil is commonly used in humans and dogs with cardiopulmonary disease, the literature on its use in cats is limited to single case reports or small case series of cats with congenital heart disease, primary respiratory disease, and parasitic disease, and limited information is available regarding the tolerability and adverse effects in cats. Given the significant benefits of sildenafil in other species, our goal was to evaluate the use of sildenafil in a larger population of cats with various cardiopulmonary diseases and to report on the tolerability and adverse effects. This retrospective study included 55 cats that received sildenafil for ≥24 h between 2009 and 2021. Forty-three cats had primary cardiac disease (acquired, *n* = 28; congenital, *n* = 15) and 12 had primary respiratory disease. Side effects possibly attributed to sildenafil included systemic hypotension (*n* = 1) and polydipsia (*n* = 1). Sildenafil was discontinued in the cat with hypotension and in three additional cats for lack of improvement in clinical signs. Median duration of sildenafil administration was 87 days (range, 2–2362 days). Sildenafil appeared to be generally well-tolerated in cats, but studies are needed to determine whether it improves the quality of life or survival times.

**Abstract:**

Sildenafil is a drug used to successfully manage a variety of cardiopulmonary disorders in people and dogs, but there is limited information on its use in cats. The objective was to review the medical records of cats that received sildenafil as part of their clinical management. Medical records and pharmacy databases were searched for cats that received sildenafil for ≥24 h between 2009 and 2021, and data were collected from medical records. Fifty-five cats received sildenafil for ≥24 h and were included in the study: 43 with primary cardiac disease (acquired, *n* = 28; congenital, *n* = 15) and 12 with primary respiratory disease. Side effects possibly attributed to sildenafil were identified in two cats (systemic hypotension, *n* = 1; polydipsia, *n* = 1), and sildenafil was discontinued in the cat with hypotension. Sildenafil was discontinued in an additional three cats due to a lack of improvement in clinical signs. No cat was documented to develop worsening pulmonary edema within 72 h of starting sildenafil. Median duration of sildenafil administration was 87 days (range, 2–2362 days). Sildenafil administration in cats appeared to be generally well-tolerated. Studies are needed to determine whether sildenafil administration to cats with cardiopulmonary disease improves the quality of life or survival times.

## 1. Introduction

Sildenafil is a phosphodiesterase type 5 (PDE5) inhibitor used to manage a variety of cardiopulmonary disorders in humans, with pulmonary hypertension (PH) and erectile dysfunction as the primary indications [1,2,3]. Inhibition of PDE5 decreases pulmonary arterial pressure (PAP) and systemic blood pressure and increases the blood flow to regional vascular beds [1,2,3]. Pulmonary hypertension is characterized by an abnormally elevated mean PAP and is associated with various diseases that cause increased pulmonary blood flow, increased pulmonary vascular resistance, or increased pulmonary venous pressure [4]. In addition to its use for PH, there has been increasing interest in the use of sildenafil in humans as a pulmonary vasodilator for congestive heart failure (CHF), based on studies showing improved symptoms, exercise tolerance, and echocardiographic parameters after sildenafil administration [3,5]. 

In the United States, the use of sildenafil in dogs and cats is an extra-label use of the drug. In dogs, there are six PH classification groups [6]. Use of sildenafil in dogs with PH is associated with an improvement in clinical signs, exercise capacity, and quality of life compared to dogs not receiving sildenafil [7,8,9,10,11]. Less information is available for PH in cats. Recently, authors have suggested that classification into six groups similar to dogs may be appropriate for cats [12].

Use of sildenafil has been described primarily in small numbers of cats with PH secondary to congenital heart disease (e.g., atrial septal defect, patent ductus arteriosus [PDA], pulmonary artery atresia, ventricular septal defects, pulmonary artery coarctation, cor triatriatum sinister) [13,14,15,16,17,18,19,20,21,22,23], restrictive cardiomyopathy [24], primary respiratory disease (e.g., pulmonary fibrosis, pulmonary capillary hemangiosis) [25,26], and parasitic diseases (e.g., heartworm, lungworm) [27,28,29]. In some of these cases, sildenafil administration appeared to improve clinical signs [13,14,17,18,19,24,28].

In addition to small numbers, previous reports on sildenafil have not included cats with other cardiovascular diseases, but it may have beneficial effects as a pulmonary vasodilator in cats with CHF that remain symptomatic despite the use of other cardiac medications. In addition, limited information is available regarding sildenafil’s tolerability and adverse effects. Given the benefits of sildenafil observed in humans and dogs with PH, our objective was to describe the use of sildenafil in a larger number of cats with various cardiopulmonary diseases and to report on the tolerability and adverse effects.

## 2. Materials and Methods

Using electronic medical records and pharmacy records at the Foster Hospital for Small Animals at Cummings School of Veterinary Medicine at Tufts University, cats treated with sildenafil for ≥24 h between 2009 and 2021 were identified for inclusion in this retrospective study. Medical records were examined to determine the cats’ underlying diseases, which were categorized as acquired heart disease (AHD), congenital heart disease (CHD), or respiratory disease (RD). Dose and frequency of sildenafil administration were recorded as well as the date sildenafil was initiated and any changes in dose or frequency. Additional medications administered to cats were recorded. For cats with cardiomyopathy, the underlying disease was classified as hypertrophic cardiomyopathy (HCM), dilated cardiomyopathy, restrictive cardiomyopathy, or cardiomyopathy with nonspecific phenotype. Physical examination findings were recorded including heart rate, respiratory rate, and the presence or absence of ascites, pleural effusion, and pulmonary edema. Evidence of pulmonary edema was documented by thoracic radiographs or confirmed via ultrasonographic findings of left atrial enlargement plus B-lines and a positive clinical response to diuretics. 

The echocardiographic data recorded included 2-dimensional and M-mode measurements, left ventricular outflow tract velocity, and tricuspid valve regurgitation velocity (TRV). In cases where these measurements were not recorded in the medical record, the original echocardiographic images were reviewed by one of the authors (JER), and measurements were performed if the images were available. Cats with cardiomyopathy were categorized into stage A, B, C, or D according to the American College of Veterinary Internal Medicine (ACVIM) consensus statement guidelines [30].

Using the ACVIM consensus statement guidelines [6], cats in the current study were classified as having a low, intermediate, or high probability of having PH. The classification was determined by examining the TRV and the structure of three anatomical locations: ventricles, pulmonary artery (PA), right atrium (RA), and caudal vena cava (CVC) [6]. Ventricles were evaluated based on the presence or absence of interventricular septal flattening, left ventricular cavity underfilling, right ventricular hypertrophy, and right ventricular cavity enlargement, and were reported as abnormal or normal based on these findings. Right ventricular hypertrophy and right ventricular cavity enlargement were graded as normal/mild, moderate, or severe. The PA diameter was measured and compared to the aortic (Ao) diameter at the same level on a right parasternal short axis view (PA:Ao). If the PA:Ao was >1, the PA was noted as enlarged and thus, abnormal. Subjective RA size from the echocardiograms was scored as normal/mild, moderate, or severe enlargement. The CVC was assessed by comparing the CVC diameter to that of the Ao, the vertebral length at the level of the carina, and width of the fourth rib [31]. Measurements for CVC assessment were obtained from lateral thoracic radiographs, or, in selected cases, from computed tomography (when lateral thoracic radiographs were unavailable or if pleural effusion obscured visualization of the CVC or Ao). Cats were assessed to have an enlarged CVC if the CVC to Ao ratio or CVC to 4th rib ratio fell within the mean ± standard error reported for cats with right heart failure from lateral thoracic radiographs (1.86 ± 0.13 and 3.12 ± 0.29, respectively) [31]. For study purposes, cats assessed to have a normal RA or CVC size were combined with cats with mild RA or CVC enlargement and categorized as having a normal RA or CVC. 

Medical records were also examined for evidence of adverse effects potentially attributable to sildenafil administration such as worsening of pulmonary edema, syncope, hypotension, lethargy, anorexia, diarrhea, vomiting, weakness, or any reason sildenafil was discontinued. If information on adverse effects was not available from the medical records, primary care veterinarians or clients were contacted to collect this information. 

Data distributions of continuous variables were evaluated using Shapiro–Wilk tests. Continuous variables are presented as median (range). Categorical variables are presented as frequency (percentages). Categorical variables were compared among disease groups (i.e., AHD, CHD, RD) using Chi square tests (or Fisher exact tests if the expected cell counts were <5). Continuous variables were compared among disease groups using ANOVA with Tukey HSD post hoc tests for normally distributed variables or Kruskal–Wallis tests with Bonferroni correction for multiple pairwise tests. All statistical analyses were performed using commercial statistical software (SPSS 28.0, IBM Corp., Armonk, NY, USA), with a *p* < 0.05 considered statistically significant.

## 3. Results

**Figure 1:**
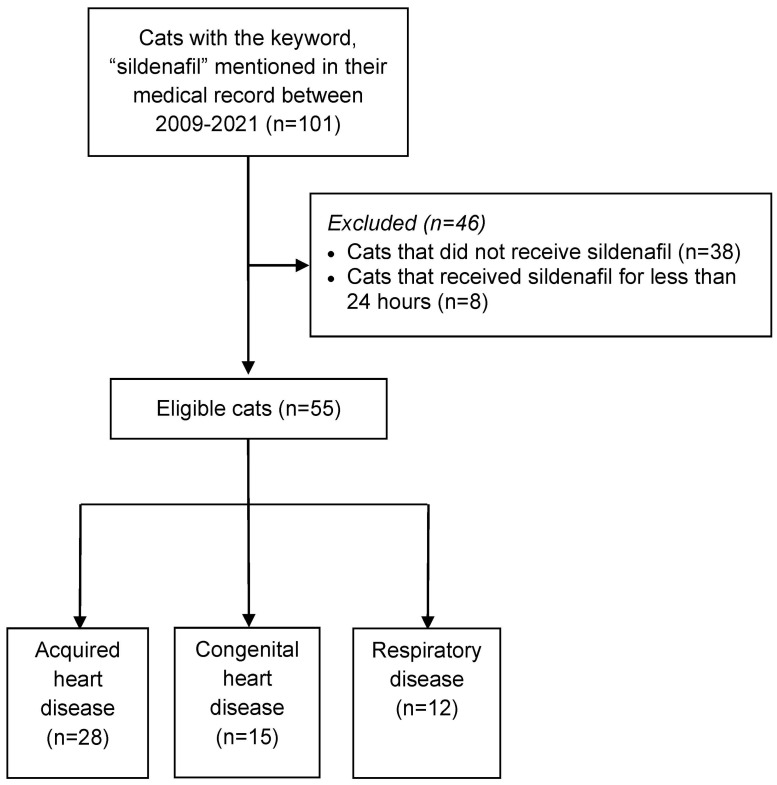
Flow diagram illustrating the enrollment of cats that received sildenafil for ≥24 h and were included in the study.

Database searches identified 101 cats with the keyword “sildenafil” mentioned in their record. Of these 101 cats, 55 received sildenafil for ≥24 h and were included in the study (Figure 1). Cats received sildenafil in tablet form (*n* = 37) or compounded form (*n* = 17 [oral suspension], *n* = 1 [capsule]). For these 55 cats, more male cats were represented (63.6%) with a median age of 10.0 years (range 0.3–18.1 years) at the time sildenafil was initiated (Table 1). Breeds included 41 domestic short- or longhair cats, Siamese (*n* = *5*), Sphynx (*n* = *4*), Persian (*n* = *2*), and one each of Abyssinian, American Shorthair, and Angora.

Disease categories for the cats included AHD (*n* = 28), CHD (*n* = 15), and RD (*n* = 12). Underlying diseases for cats in the AHD group included HCM (*n* = 21), cardiomyopathy with nonspecific phenotype (*n* = 4), restrictive cardiomyopathy (*n* = 2), and dilated cardiomyopathy (*n* = 1) with cats in stage C (*n* = 10) and stage D (*n* = 18). Of the 15 cats in the CHD group, 11 cats had a right-to-left shunting defect (ventricular septal defect, *n* = 5; PDA, *n* = 4; ventricular septal defect and PDA, *n* = 1; and atrial septal defect, *n* = 1). The remaining CHD diagnoses included mitral valve stenosis (*n* = 2), supravalvular mitral stenosis (*n* = 1), and cor triatriatum sinister (*n* = 1). Twenty-eight out of twenty-eight cats in the AHD (100%) and 9/15 cats in the CHD groups (60%) had CHF. Cats in the CHD group were significantly younger than cats with AHD or RD (*p* < 0.001; Table 1) and weighed less than cats with AHD (*p* < 0.001). Sex distribution was not significantly different among the three disease groups. There was no significant difference in the initial sildenafil dose among the three disease groups, with the median dose for all cats of 3.3 mg/kg/day (range, 0.8–8.0 mg/kg/day). Concurrent medications were common in this population of cats (Table 1).

Thirty cats had a measurable TRV. Tricuspid valve regurgitation velocity at diagnosis was significantly higher in the CHD and RD groups compared to the AHD group (*p* < 0.001; Table 2), with the CHD group having the highest velocity (median = 4.55 m/s [range, 3.30–5.95 m/s]) followed by the RD group (median = 3.64 m/s [range, 2.43–4.98 m/s]) and the AHD group (median = 2.88 m/s [range, 1.25–3.31 m/s]). Based on the ACVIM consensus statement criteria for dogs [6], 56.6% of cats had a high probability of PH, 26.4% had intermediate probability, and 17.0% had low probability. Disease groups were significantly different in PH probability (*p* = 0.02; Table 2). Cats in the CHD group had mostly high probability for PH (80.0%), while 75.0% of cats in the RD group had high probability, and 34.6% of cats in the AHD group had high probability (Table 2). There was a significant difference in PH probability between the CHD and AHD groups (*p* = 0.01), with no significant difference between the RD and either the CHD (*p* = 0.27) or AHD groups (*p* = 0.06). 

Sildenafil doses were increased in seven cats (AHD, *n* = 5; CHD, *n* = 2; Table 1) for a final dose for all cats of 3.4 mg/kg/day (0.8–12.5 mg/kg/day) with no significant difference among disease groups. Many cats received concurrent medications including furosemide (*n* = 39), pimobendan (*n* = 35), clopidogrel (*n* = 29), and an angiotensin converting enzyme inhibitor (*n* = 19); Table 1). The median duration of sildenafil administration was 87 days (range, 2–2362 days), but was not significantly different among the three groups (Table 1). 

Side effects potentially attributable to sildenafil occurred in two cats: systemic hypotension (*n* = 1) and polydipsia (*n* = 1). Sildenafil was discontinued in the cat with hypotension. Sildenafil was discontinued in an additional three cats, with owners citing a lack of improvement as the primary reason for stopping the medication. No cat was documented to develop worsening pulmonary edema within 72 h of starting sildenafil. One cat with active pleural effusion and prior pulmonary edema developed worsening dyspnea two days after starting sildenafil, and dyspnea resolved after thoracentesis and discontinuation of sildenafil. Three cats with pleural effusion previously requiring thoracentesis were presented for dyspnea, and repeat thoracentesis was performed four to six days after starting sildenafil.

At the time of analysis, three cats were still alive (all in the CHD group); two of these three cats were still receiving sildenafil. Of the remaining 52 cats, most were euthanized (*n* = 28) or died (15 total [CHF, *n* = 7; sudden death, *n* = *4*; respiratory distress unrelated to CHF, *n* = 1; aortic thromboembolism, *n* = 1; anesthetic death, *n* = 1; trauma, *n* = 1]). Three cats were confirmed to be deceased but the cause was unknown, and six were lost to follow-up. 

## 4. Discussion

Most cats (78%) that received sildenafil in the current study had either acquired or congenital heart disease; 22% had respiratory disease. Most cats (83%) had intermediate or high probability of having PH (100% in the CHD group, 83% in the RD group, and 73% in the AHD group). Sildenafil is most commonly used in dogs and cats to treat PH. Positive clinical and echocardiographic findings have been noted with sildenafil administration in dogs with PH [7,11]. In dogs with bidirectional and continuous right-to-left shunting PDA, sildenafil administration was associated with a longer survival time [20]. In cats, improvements in respiratory signs, exercise capacity, and/or echocardiographic measurements were noted with the addition of sildenafil to treat PH [13,14,17,18,19,24,28]. The most common diseases in cats for which sildenafil was used in prior case reports were similar to those in the current study including PH secondary to CHD, AHD, and RD [13,14,15,16,17,18,19,20,21,22,23,24,25,26]. In this current study, PH secondary to parasitic disease was not documented in any cat. 

More recently, sildenafil has been used to treat humans with CHF not associated with PH, but sildenafil is not yet approved for this indication [1,3,5,32]. In humans with CHF with reduced ejection fraction caused by dilated or ischemic cardiomyopathy, sildenafil administration was associated with improved symptoms of dyspnea and orthopnea, exercise tolerance, and echocardiographic parameters [3]. Additionally, sildenafil administration is being evaluated in humans for the treatment of heart failure with preserved ejection fraction [32], which raises the possibility of using sildenafil for feline cardiomyopathies such as HCM. In the current study, HCM was the most common acquired heart disease, accounting for 21/28 cats (75%) in the AHD group.

There was a wide range of the initial sildenafil dose used in the current study (0.8–8.0 mg/kg/day). This dose range is similar to doses used in previous reports in cats, which have ranged from 0.5 to 6 mg/kg/day [13,19,33]. Sildenafil appeared to be generally well-tolerated in cats, with only two cats showing possible side effects of sildenafil: one cat with polydipsia and one with systemic hypotension. The cat with polydipsia was also receiving furosemide, the dose of which was increased at the same time sildenafil was initiated; sildenafil was continued. Sildenafil was discontinued in the cat with hypotension. In a prior report, one cat with a pulmonary vasoproliferative process had a presumed adverse reaction to sildenafil with sudden onset respiratory distress shortly after sildenafil administration [26]. In that cat, the hypothesis was that sudden onset pulmonary edema developed after sildenafil administration [26]. No cat in the current study was documented to develop worsening pulmonary edema within 72 h of starting sildenafil, although this is an important potential side effect to evaluate prospectively in future studies and with clinical use of sildenafil in cats. Reported side effects in dogs following sildenafil administration have included transient diarrhea [34,35], transient hyporexia [36], flushing of the inguinal region [7], vomiting [35], lethargy, somnolence, clear nasal discharge, and erect ears [8]. Owners of three cats in the current study discontinued sildenafil due to a perceived lack of improvement in clinical signs. 

Cat-specific criteria for the diagnosis and classification of PH are not available, so we elected to use the ACVIM consensus guidelines for PH in dogs in the current study [6]. Ideally, right heart catheterization and PAP measurement are needed for the diagnosis of PH. However, this is invasive, expensive, and not feasible in this patient population where most cats presented with respiratory distress. Thus, PH probability was assessed by echocardiographic data using TRV as a surrogate measurement of PAP and assessment of the three anatomical locations commonly affected by altered pulmonary hemodynamics via echocardiography and other diagnostic imaging approaches [6]. Most cats in the current study had intermediate or high probability of PH, with only 17% having low probability of PH. Two previous studies have attempted to apply the same ACVIM consensus guidelines to cats with hyperthyroidism [37,38]. In both studies, TRV was unmeasurable in most of the cats (≥90% in both studies) [37,38]. In the current study, 30 of the cats (55%) had a measurable TRV. Missing TRV measurements may be secondary to a lack of tricuspid regurgitation or to an incomplete echocardiographic examination, which can be due to the temperament or stability of the patient. 

Although using the ACVIM consensus statement guidelines for PH in dogs [6] is feasible, it represents an important limitation of this study. Translating canine methods and specific values to cats can be fraught with pitfalls and the same criteria for diagnosis and categorization of PH may not be applicable to cats. Therefore, the TRV cutoff of 3.4 m/s and other criteria from the ACVIM consensus statement for dogs may not be appropriate for the diagnosis of PH in cats. Since echocardiography-based criteria are preferable for clinical use, evidence-based, cat-specific guidelines on echocardiographic techniques and reference parameters for PH diagnosis are needed. 

There are other important limitations of the study. Because of the retrospective design, some data points were missing due to incomplete medical records or the lack of follow-up. The relatively small sample size, especially in the CHD and RD groups, limited the statistical power. This may have resulted in type II errors for some comparisons. Additionally, this study was conducted at a tertiary referral center, so this population may not reflect the general cat population. Since many cats were at an advanced stage of disease and were not stable outside of the oxygen cage at the time, a decision was made to initiate sildenafil. Complete echocardiograms could not always be performed and relatively few cats had a subsequent echocardiogram to assess their response to sildenafil administration. As a result, there were few cats with a comparison of pre- and post-sildenafil TRV. Finally, since most cats had advanced disease at the time sildenafil was initiated, most were taking other medications in addition to sildenafil, which could complicate the interpretation of adverse effects, efficacy, and outcome. 

## 5. Conclusions

Based on these results, sildenafil administration in cats is generally well-tolerated. Future studies are indicated to determine whether sildenafil administration to cats with cardiopulmonary disease results in improved quality of life or survival times. Although the ACVIM consensus statement on PH in dogs appeared to be useful for the classification of the probability of PH in cats, further investigation is needed to confirm the best approaches to document PH in cats. 

## Figures and Tables

**Table 1:** Summary of signalment, physical examination findings, and medications in 55 cats that received sildenafil between 2009 and 2021. Data are presented as median (range) or frequency (percent). The number of cats (*n*) for each variable is shown if not all cats had information available for that variable. Results within a row with different superscript letters were significantly different from one another, with a *p* < 0.05 considered statistically significant. For example, for body weight, *p* < 0.001 in the *p*-value column indicates there was a significant difference among the three disease groups. The superscript letters in the body weight row show that cats in the congenital heart disease group (superscript b) weighed significantly less than the cats in the acquired heart disease group (superscript a), but that cats in the respiratory disease group (superscript ab) were not significantly different from either the acquired or congenital heart disease groups.

	Disease Group			Cats from All 3 Disease Groups Combined	*p*-Value
	Acquired Heart Disease	Congenital Heart Disease	Respiratory Disease		
*n*	28	15	12	55	
Signalment					
Body weight (kg)	4.5 (2.6–7.8) ^a^	3.2 (1.0–4.6) ^b^	4.1 (2.8–5.5) ^ab^	4.0 (1.0–7.8)	<0.001
Age (years)	12.5 (1.3–18.1) ^a^	1.0 (0.3–16.9) ^b^	10.1 (2.1–15.8) ^a^	10.0 (0.3–18.1)	<0.001
Sex					0.44
Male	19 (18 castrated)	9 (8 castrated)	7 (6 castrated)	35 (32 castrated)	
Female	9 (9 spayed)	6 (4 spayed)	5 (5 spayed)	20 (18 spayed)	
Physical examination findings					
Respiratory rate (per minute)	40 (24–84) ^a^	55 (32–84) ^b^	39 (20–80) ^a^	40 (20–84)	0.01
	*n* = 23	*n* = 12	*n* = 10	*n* = 45	
Heart rate (per minute)	200 (120–250) ^ab^	212 (150–240) ^a^	180 (140–216) ^b^	200 (120–250)	0.02
	*n* = 23	*n* = 13	*n* = 10	*n* = 46	
Sildenafil details					
Sildenafil initial dose (mg/kg/day)	3.1 (1.8–4.7)	3.8 (0.8–8.0)	3.9 (1.8–6.2)	3.3 (0.8–8.0)	0.09
Number of cats in which the sildenafil dose was increased	5 (17.9%)	2 (13.3%)	0 (0.0%)	7 (12.7%)	0.38
Sildenafil final dose (mg/kg/day)	3.1 (1.8–9.4)	4.6 (0.8–12.5)	3.8 (1.8–6.2)	3.4 (0.8–12.5)	0.37
Sildenafil duration (days)	101 (2–1834)	223 (2–2362)	12 (2–158)	87 (2–2362)	0.17
	*n* = 26	*n* = 12	*n* = 6	*n* = 44	
Concurrent medications					
Furosemide	28 (100.0%) ^a^	9 (60.0%) ^b^	2 (16.7%) ^c^	39 (70.9%)	<0.001
Furosemide mg/kg/day	3.5 (0.7–13.7)	3.9 (0.7–5.9)	1.9 (1.6–2.2)	3.5 (0.7–13.7)	0.46
ACE inhibitor	15 (53.6%) ^a^	4 (26.7%) ^ab^	0 (0.0%) ^b^	19 (34.5%)	0.004
Clopidogrel	22 (78.6%) ^a^	3 (20.0%) ^b^	4 (33.3%) ^b^	29 (52.7%)	<0.001
Pimobendan	25 (89.3%) ^a^	6 (40.0%) ^b^	4 (33.3%) ^b^	35 (63.6%)	<0.001

Abbreviation: ACE, angiotenson converting enzyme.

**Table 2:** Summary of the American College of Veterinary Internal Medicine consensus statement [6] criteria for pulmonary hypertension in dogs and associated imaging parameters with results for 55 cats that received sildenafil. Data are presented as median (range) or frequency (percentage). The number of cats (n) for each variable is shown if not all cats had information available for that variable. Results within a row with different superscript letters were significantly different from one another, with a *p* < 0.05 considered statistically significant. For example, for tricuspid regurgitation velocity, *p* < 0.001 in the *p*-value column indicates there was a significant difference among the three disease groups. The superscript letters in the tricuspid regurgitation velocity row show that cats in the congenital heart disease group (superscript b) and respiratory disease groups (superscript b) had a higher tricuspid regurgitation velocity than cats in the acquired heart disease group (superscript a). Cats in the congenital and respiratory disease groups had the same superscript letter (b), which indicates that there was not a significant difference between these two groups.

Anatomic Locations	Disease Groups			Cats from All 3 Disease Groups Combined	*p*-Value
	Acquired Heart Disease	Congenital Heart Disease	Respiratory Disease		
*n*	28	15	12	55	
Tricuspid regurgitation velocity (m/s)	2.88 (1.25–3.31) ^a^	4.55 (3.30–5.95) ^b^	3.64 (2.43–4.98) ^b^	3.39 (1.25–5.95)	<0.001
	*n* = 12	*n* = 10	*n* = 8	*n* = 30	
Tricuspid regurgitation velocity					<0.001
<3.0 m/s or not measurable	7/12 (58.3%)	0/10 (0.0%)	1/8 (12.5%)	8/30 (26.7%)	
3.0–3.4 m/s	5/12 (41.7%)	1/10 (10.0%)	1/8 (12.5%)	7/30 (23.3%)	
>3.4 m/s	0/12 (0.0%)	9/10 (90.0%)	6/8 (75.0%)	15/30 (50.0%)	
Right atrial or caudal vena cava enlargement	15/26 (57.7%)	9/15 (60.0%)	10/12 (83.3%)	34/53 (64.2%)	0.29
Abnormal ventricles	19/26 (73.1%)	15/15 (100.0%)	10/12 (83.3%)	44/53 (83.0%)	0.07
Abnormal pulmonary artery	13/22 (59.1%) ^a^	10/12 (83.3%) ^ab^	10/10 (100.0%) ^b^	33/44 (75.0%)	0.03
Pulmonary hypertension likelihood					0.02
Low	7/26 (26.9%)	0/15 (0.0%)	2/12 (16.7%)	9/53 (17.0%)	
Intermediate	10/26 (38.5%)	3/15 (20.0%)	1/12 (8.3%)	14/53 (26.4%)	
High	9/26 (34.6%)	12/15 (80.0%)	9/12 (75.0%)	30/53 (56.6%)	

## Data Availability

Dataset available upon reasonable request from the authors.
